# Geospatial Interpolation and Mapping of Tropospheric Ozone Pollution Using Geostatistics

**DOI:** 10.3390/ijerph110100983

**Published:** 2014-01-10

**Authors:** Swatantra R. Kethireddy, Paul B. Tchounwou, Hafiz A. Ahmad, Anjaneyulu Yerramilli, John H. Young

**Affiliations:** 1 Trent Lott Geospatial and Visualization Research Center, College of Science Engineering and Technology, Jackson State University, Mississippi E-Center, 1230 Raymond Rd, Jackson, MS 39204, USA; E-Mails: swatantra.r.kethireddy@students.jsums.edu (S.R.K.); anjaneyulu.yerramilli@jsums.edu (A.Y.); john.h.young@jsums.edu (J.H.Y.); 2 NIH RCMI Center for Environmental Health, Jackson State University, 1400 JR Lynch Street, P.O. Box 18750, Jackson, MS 39217, USA; 3 Department of Biology, Jackson State University, 1400 JR Lynch Street, Jackson, MS 39217, USA; E-Mail: hafiz.a.ahmad@jsums.edu

**Keywords:** tropospheric ozone (O_3_), geostatistical analysis, prediction, interpolation, spatial resolution, visualization, Geographical Information Systems (GIS)

## Abstract

Tropospheric ozone (O_3_) pollution is a major problem worldwide, including in the United States of America (USA), particularly during the summer months. Ozone oxidative capacity and its impact on human health have attracted the attention of the scientific community. In the USA, sparse spatial observations for O_3_ may not provide a reliable source of data over a geo-environmental region. Geostatistical Analyst in ArcGIS has the capability to interpolate values in unmonitored geo-spaces of interest. In this study of eastern Texas O_3_ pollution, hourly episodes for spring and summer 2012 were selectively identified. To visualize the O_3_ distribution, geostatistical techniques were employed in ArcMap. Using ordinary Kriging, geostatistical layers of O_3_ for all the studied hours were predicted and mapped at a spatial resolution of 1 kilometer. A decent level of prediction accuracy was achieved and was confirmed from cross-validation results. The mean prediction error was close to 0, the root mean-standardized-prediction error was close to 1, and the root mean square and average standard errors were small. O_3_ pollution map data can be further used in analysis and modeling studies. Kriging results and O_3_ decadal trends indicate that the populace in Houston-Sugar Land-Baytown, Dallas-Fort Worth-Arlington, Beaumont-Port Arthur, San Antonio, and Longview are repeatedly exposed to high levels of O_3_-related pollution, and are prone to the corresponding respiratory and cardiovascular health effects. Optimization of the monitoring network proves to be an added advantage for the accurate prediction of exposure levels.

## 1. Introduction

Air pollution is a spatial and temporal phenomenon. In the past few decades, though high levels of ozone (O_3_) pollution have been recorded in intense traffic areas and industrialized cities, their characteristics and impacts were also observed in rural areas. Therefore, O_3_ pollution could be trans-boundary in nature [1]. Recently, as more sensitive instrumentation to monitor O_3_ concentrations is in use, it became clear that O_3_ pollution is not limited to urban environments but can extend to regional and global scales in the troposphere [2,3]. Due to wind flow, fluctuations in atmospheric conditions, and regionally varying temperatures, O_3_ precursors in the troposphere cross states to remote areas within a short time. Morris *et al.* have conducted a study to demonstrate the impact of Alaskan and Canadian forest fires on O_3_ levels in Houston, Texas. They were able to establish the relation using the National Aeronautics and Space Administration (NASA) Goddard trajectory model, Earth Probe satellite data, Moderate Resolution Imaging Spectroradiometer (MODIS) Terra data, and *in situ* measurements [3]. Another study by Stutz *et al.* used Differential Optical Absorption Spectroscopy (DOAS) measurements to study the vertical distributions of O_3_, NO_2_, and NO_3_ near Houston, TX. They concluded that the chemistry in polluted areas is strongly altitude dependent in the lowest 100 meters of the nocturnal atmosphere [4]. Recently, a research conducted by Banta *et al.* has exploited the capabilities of O_3_-profiling differential absorption lidar (DIAL) to map O_3_ pollution in Houston; the analysis was useful for documenting meteorological ingredients needed to accurately predict high pollution events [5]. A field campaign study by Cohan *et al.* concluded that the reduction in NO_X_ and highly reactive volatile organic compounds had large contributions on ozone levels, which declined by 40–50% [6]. Visual understanding of O_3_ pollution, its nature, behavior and interaction with populations and the environment are of high interest in this context and are crucial from a geospatial and health perspective. Although dispersion of pollutants in the troposphere is a complex process, researchers are making considerable efforts to simplify this complexity in a number of ways and understand the characteristics of their distribution over time [7]. A paper published by Brody *et al.* suggests that the public perceptions of air quality in Texas are driven not by the actual air quality measurements, and instead the perceptions are formed by factors such as sense of place, neighborhood setting, source of pollution, and socioeconomic characteristics. The authors used a GIS-based spatial and statistical approach to examine localized patterns of air quality perception in Texas [8]. 

Ground level O_3_ studies are of great interest among environmental scientists and air quality professionals as well as among regulatory agencies [9,10,11]. Effective decision making has a large impact on public health, and an accurately predicted pollution map is a significant source of information for the decision maker. The map accuracy depends on a reliable dense network of monitors in a study region and the model parameters used to produce a statistically valid pollution map [12]. In contrast to the present study, Anjaneyulu *et al.* have successfully employed a WRF/Chem model to simulate urban microscale surface ozone levels for Jackson, MS at 1 kilometer spatial resolution [13]. 

Technological and scientific advances have led to development of geospatial platforms in which certain tools and extensions allow us to study the spatio-temporal changes of geo-environmental phenomena. ArcGIS 10.1 was developed by ESRI^®^ (Environmental Systems Research Institute, Redlands, CA, USA). Geostatistical tools in ArcGIS help to exploit the statistical properties of data, understand, interpret, and make decisions based on the analysis [14]. The main advantage is visual understanding of analysis which gains the attention of the map user. Moroko *et al.* pointed out that robust understanding of ongoing phenomenon is only possible when data are exploited, analyzed, and mapped by suitable techniques [15]. It has been reported that geostatistical analysis can assess potential environmental hazards by interpolating the possible flow and direction of air pollution, biohazard releases, and any potential harmful waste that may be introduced into areas of human habitation [16]. 

A unique quality of this study was to use the multi-disciplinary approach of geospatial visual analytics, geostatistics, and atmospheric science to address the problem of Texas state air pollution. The main objectives of this study are to: (1) analyze and map the selected hourly episodes of O_3_ concentrations for spring and summer 2012 over eastern Texas; (2) predict and validate O_3_ concentrations for unmonitored geo-spaces; and (3) measure the intensity of O_3_ pollution in the study area. 

## 2. Experimental Section

### 2.1. Description of the Study Area

**Figure 1:**
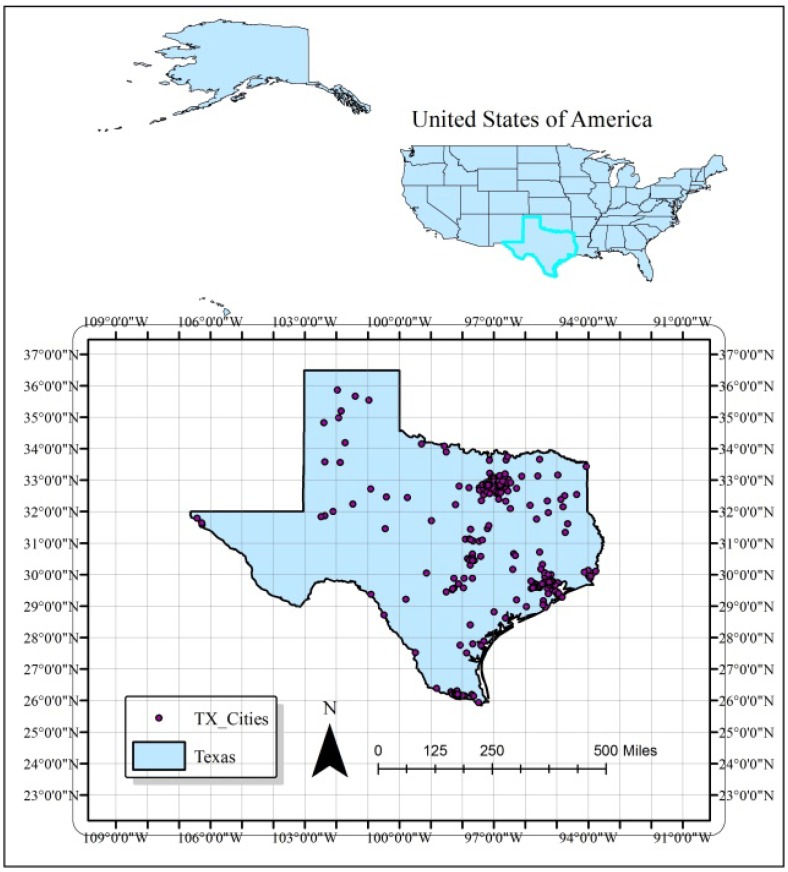
Location of Texas and its cities.

**Figure 2:**
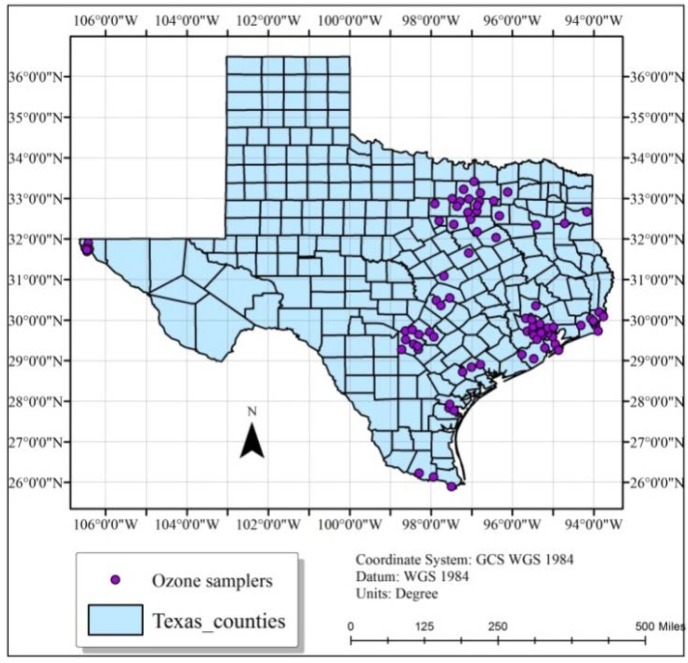
Location of O_3_ sampling stations in Texas.

Texas, the second largest state in the US, is located in the south central region. It has a land area of 261,231.7 square miles with a population of 25,145,561 according to the 2010 census [17]. It is the number one total energy producer in the nation and the sixth in total energy consumed per capita as of 2010 [18]. Due to increased urbanization and industrial activities (crude oil refineries, chemical and coal fired power plants), the state is very prone to air pollution. Figure 1 represents the State of Texas showing the spatial distribution of its cities which are mainly concentrated in the eastern part. During the last decade, Texas recorded bad air quality in 2011 [19]. A review of TAMIS-Texas Air Monitoring Information System data indicated that a similar trend during the summer of 2012, despite stringent laws and air pollution regulations. The state has 80 to 100 O_3_ sampling stations (numbers may vary due to maintenance problems). Texas has heavy traffic, and shares a boundary with Mexican states (air pollutants have no boundaries). As air pollution is mostly an urban phenomenon, most of the sampling stations are scattered around the eastern part of the state, and in major cities such as Dallas, Houston, Austin, and San Antonio. Therefore, the air quality is of major concern in this region. Pollution monitoring stations are non-uniformly distributed on the ground and O_3_ sampling stations are sparse in the western half of state (Figure 2). In order to obtain significant and statistically meaningful results, geostatistical analysis and interpolation were carried out only in the eastern half of state.

### 2.2. Data Sources and Software

Hourly O_3_ pollution data were obtained from the TAMIS. The raw data were initially preprocessed and converted into a database file (DBF) using Microsoft Access software and then imported into ArcGIS for analysis and mapping. Data were integrated with geographical reference information which is a critical requirement for geospatial data. Background map data were obtained from ESRI^®^ ArcGIS online resources and Texas hill shade map data were obtained from the Texas Water Development Board website. 

### 2.3. Methodology

Geostatistics functionality applies to regionalized phenomena both natural and manmade. It assumes the phenomena that occur in Nature to be spatially dependent or correlated. Miller reported that Tobler’s first law of geography is the core of spatial interpolation and geostatistical analysis [20]. Samples taken at nearby locations are expected to have more similar values than samples taken farther apart, Tobler said that everything is related to everything else [21]. A few examples are ocean salinity and air pollution. In the United States, thousands of O_3_ monitoring stations are scattered throughout the States. In most cases they are distributed in urban areas. In practicality, it is impossible to establish stations in each place of interest due to economic considerations and other restrictions. But quantification at unmeasured locations is important to understand how intense the pollution is. Geostatistical techniques assume the existence of spatial correlations in sampled values of O_3_. Spatially correlated values not only facilitate optimal mapping of the pollution in the entire area but also provide valuable information about the air quality of that area. The objective of spatial interpolation is to predict the air pollution of a region by estimating the concentrations at unmeasured locations based on values at measured locations. Diem advised that O_3_ mapping projects must report spatial scale, provide the spatial resolution of the produced map, include a scale bar, and present the statistical accuracy of the generated map [10]. ArcGIS Geostatistical Analyst allows us to create a statistically valid prediction surface along with prediction uncertainties from a limited number of data points [14].

Kriging is a geostatistical method first used in mining and geological engineering in the 1950s [22]. Since then, it has been used in air quality studies [7,23,24,25]. Fraczek *et al.* said that the intensity of phenomenon can be accurately predicted and estimated by the Kriging technique. The main advantage of using Kriging in spatial interpolation is its ability to calculate the uncertainty of prediction which is useful in decision making. A Kriging Interpolation model predicts surfaces better than other models when data are checked for outliers and errors [14]. If the data follow a normal distribution, Kriging is the best unbiased method of predicting a surface [14]. However, spatial prediction does not require the data to be normally distributed, because Kriging is the best linear unbiased method of predicting a surface [26]. 

#### 2.3.1. Exploration of Spatial Data

Whenever mean and median values appear close to each other, it may be evidence of normality in the data. For better understanding the data, ArcGIS offers a set of tools by which we can explore the distribution, identify global trends, and calculate the spatial auto correlation in the data. Two tools that were used to check outliers and normality of O_3_ data are: (1) Histogram, (2) Normal Quantile-Quantile (QQ) plot:

(1) Use of histogram to represent the distribution:
  O_3_ values were split into ten classes (X-Axis values were redrawn to a scale of 10). In each class, the frequency was represented by the height of the bar.
(2) Use of QQ plot to compare the data distribution to the standard normal distribution:
  Data are said to be normal if they follow a “45°” straight line. For all the studied hours, QQ plots were drawn in which quantiles of O_3_ data were plotted against the quantiles of the standard normal distribution.

#### 2.3.2. Global Trends in Data

Plotting the values in three dimensional spaces provides location based information about the data. In trend analysis graphs, the height of each vertical line is the measured value at that particular location. Data values were represented as perpendicular planes in North-South and the East-West planes. Two best fit polynomials were drawn for thorough understanding, one in the north-south (blue line) and the other in East-West (green line) direction. A U shaped curve in polynomials indicates the presence of a trend, if any. If there were no trends, the blue and green lines would appear flat in 3D space. 

#### 2.3.3. Spatial Correlation in Data

The Kriging method uses semivariance to measure spatial correlation in sampled values. A semivariogram measures the strength of statistical correlation in measured values as a function of distance. It assumes the values that are close to each other in space are more alike. The existence of spatial dependence in a dataset can be found by small semivariance in data points that are close to each other and larger semivariances in data points that are farther apart [21]. Semivariance is computed by the following equation:

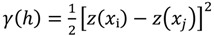
(1)
where *γ*(*h*) is semivarience between known data points, x_i_ and x_j_, separated by a distance h, and z is the attribute value. For all the studied hours of semivariogram plots, the difference squared between values of each pair of locations was plotted on the y-axis and the distance separating each pair of those measurement values was plotted on the x-axis. The equation to calculate average semivariance is:

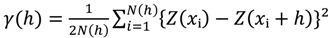
(2)
where *γ*(*h*) is the average semivariance between sample points separated by distance h, n is the numbers of pairs of sample points, and z is the attribute value. 

#### 2.3.4. Cross Validation

The objective of cross validation is to determine how well the model has predicted unknown values (unmonitored areas). The method sequentially omits a data point and calculates the predicted value from the remaining database for that particular location. The difference between measured and predicted values is called prediction error. Statistics are calculated on prediction errors, and the accuracy of produced pollution maps is assessed based on standard criteria. The accuracy of predicted values depends on three parameters [14] including unbiased predictions, accurate standard errors, and closeness of predicted values to measured values.

## 3. Results and Discussion

### 3.1. Normality and Checking for Errors

#### 3.1.1. Histogram

**Figure 3:**
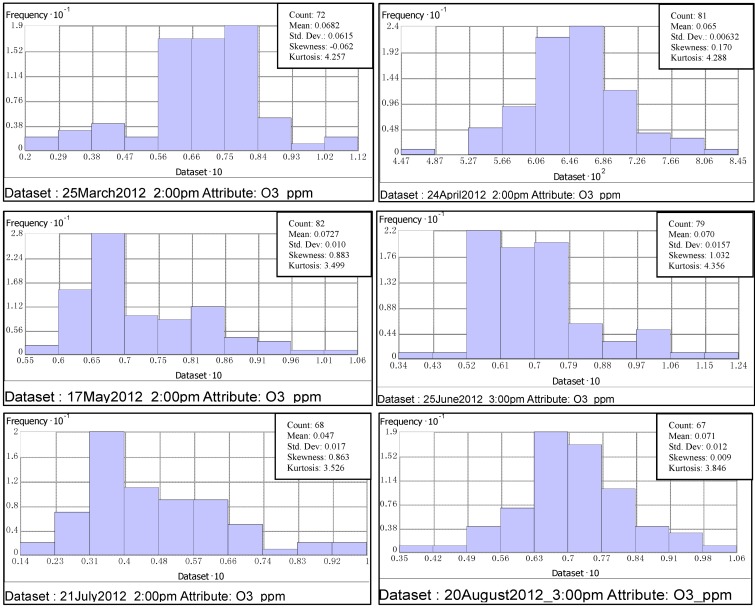
Representation of O_3_ data for the studied hours.

From the data presented in Figure 3, the distribution of O_3_ concentrations was insignificantly skewed either right or left. Normality is indicated not only by the histograms but also by the closeness of mean and median values of ozone concentrations presented in Figure 3. There was a low frequency of high concentration values. For all the studied hours of spring and summer 2012 days, a noticeable fact is that the highest O_3_ concentrations (>0.08 ppmv) clustered on the right side of histogram were always in urban areas (North-East and South-East regions of the State). Except For 21 July 2012 at 2:00 pm, high frequency values that fell between 0.55 ppmv and 0.75 ppmv were randomly distributed throughout the Eastern part of the state. 

#### 3.1.2. Quantile-Quantile Plot

The other way of studying and understanding the data is by normal QQ plots; data points appear close to the “45°” line and close enough to indicate normality. Deviations from a straight line have been observed at the lowest and highest O_3_ values. During data exploration and analysis, it was visualized and clearly understood that the highest O_3_ values (>0.08 ppmv) fell in the North-East, and South-East regions; details are presented in Table 1. Ozone pollution is mostly confined to them and distributed in and around those regions. In the case where data are not normally distributed, different types of data transformation techniques (Box-Cox, logarithmic, and arcsine) can be applied to make them normal [27]. 

**Table 1:** Regions of Eastern Texas in which concentrations of O_3_ > 0.08 ppmv for the studied hours.

25 March 2012 at 2:00 pm	24 April 2012 at 2:00 pm	17 May 2012 at 2:00 pm	25 June 2012 at 3:00 pm	21 July 2012 at 2:00 pm	20 August 2012 at 3:00 pm
Dallas/Fort Worth, Houston, and Beaumont	Houston and Dallas/Fort Worth	Houston, Austin, Dallas/Fort worth, San Antonio, Beaumont, and Corpus Christi	Dallas/Fort Worth, Houston	Dallas/Fort Worth	Dallas/Fort Worth, Houston, and San Antonio

### 3.2. Global Trend Analysis

**Figure 4:**
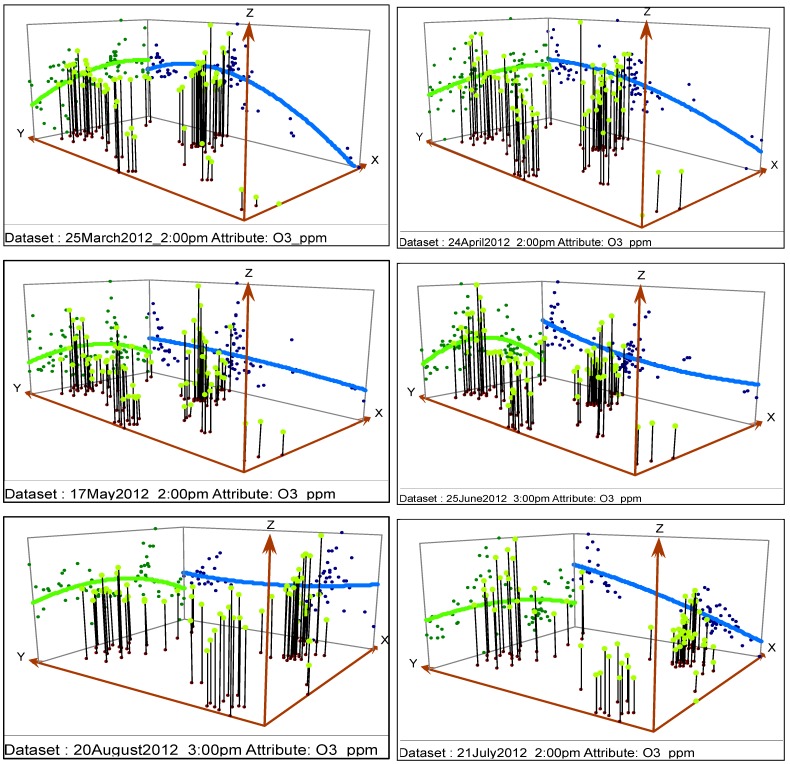
Representation of O_3_ data for the studied hours.

Careful observation of polynomials presented in Figure 4 indicates that the lines start at low values and then increase in trend as they move to the North-East region. Except for 20 August 2012 at 3:00 pm, the trend of the blue line always increases as it moves from South to North. Except for 25 March 2012 at 2:00 pm and 24 April 2012 at 2:00 pm, the green polynomial increases as it moves East and then decreases from the center of the domain, indicating more O_3_ pollution in the North Central region for those hours. Table 1 lists the areas more vulnerable to O_3_ pollution where monitoring activities have already been implemented.

### 3.3. Spatial Correlation

**Figure 5:**
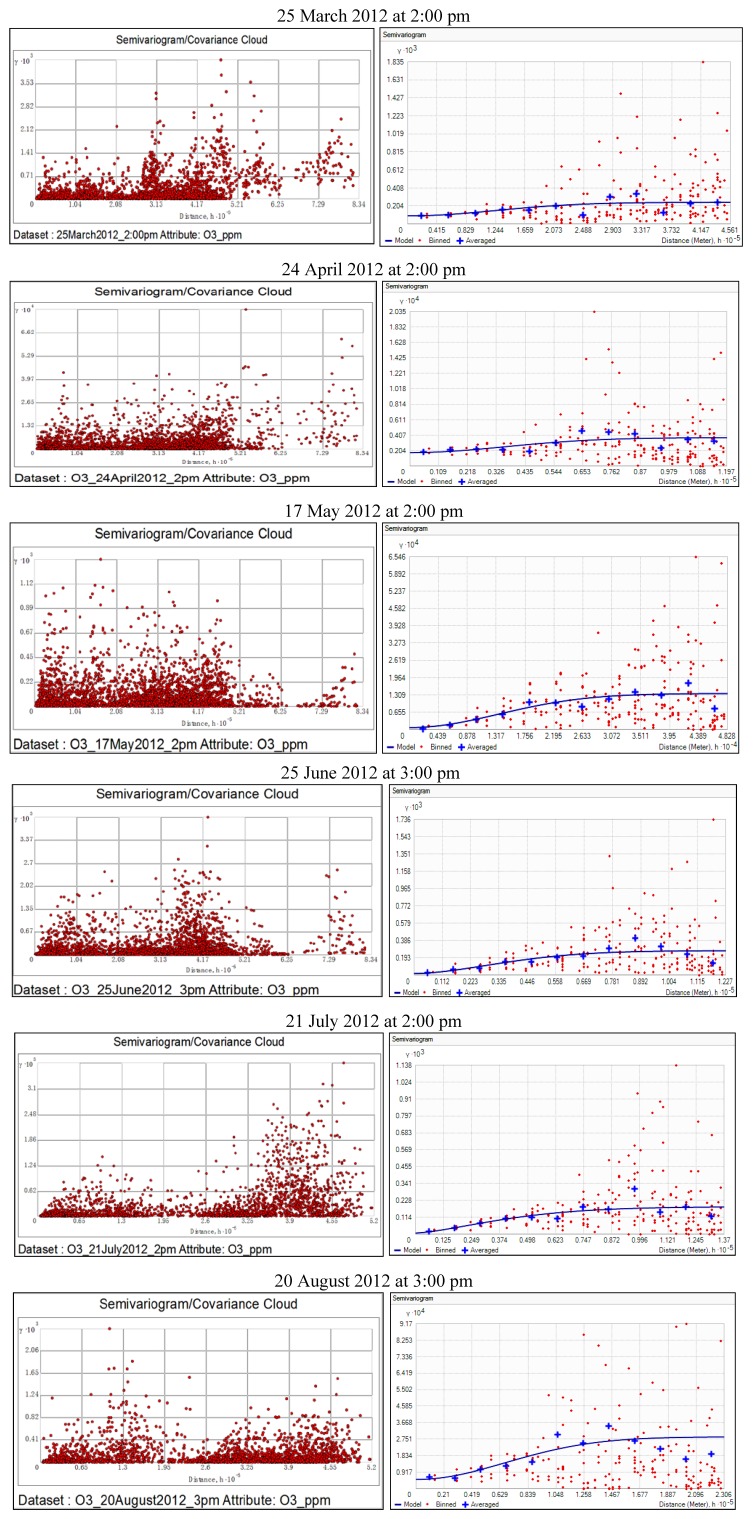
Spatial autocorrelation in O_3_ data for the studied hours.

According to the principles of geostatistics, locations of data that are close to each other should be similar. As the distance between the locations increases, the difference between corresponding data should also increase. In the semivariogram cloud plots shown in Figure 5, each red dot represents a pair of sampling locations. Each pair of locations that are close to each other (left on x-axis) had a small semivariance (low values on y-axis), and as the distance increased (moving right on x-axis), the semivariance values also increased (high values on y-axis). The graphs below represent a picture of the spatial correlations in data for the hours examined. The best fit lines were determined by regression analysis. The pairs of locations (each red point in the cloud) were grouped into separation distance classes (lags). In ArcGIS, the geostatistical analyst extension tool was used to construct stable semivariogram models for the studied hours using ordinary Kriging. It was observed that the blue line rose at smaller distances and flattened off at larger distances, indicating that there is little autocorrelation in O_3_ data at larger distances. Because of their wide application to environmental data analysis [14], omnidirectional stable semivariogram models were constructed using optimal parameter values (nugget, range, partial sill, and shape). However, there are several other types of semivariogram models (circular, spherical, exponential, and Gaussian *etc.*) that could be used depending on how well they fit the data. 

**Figure 6:**
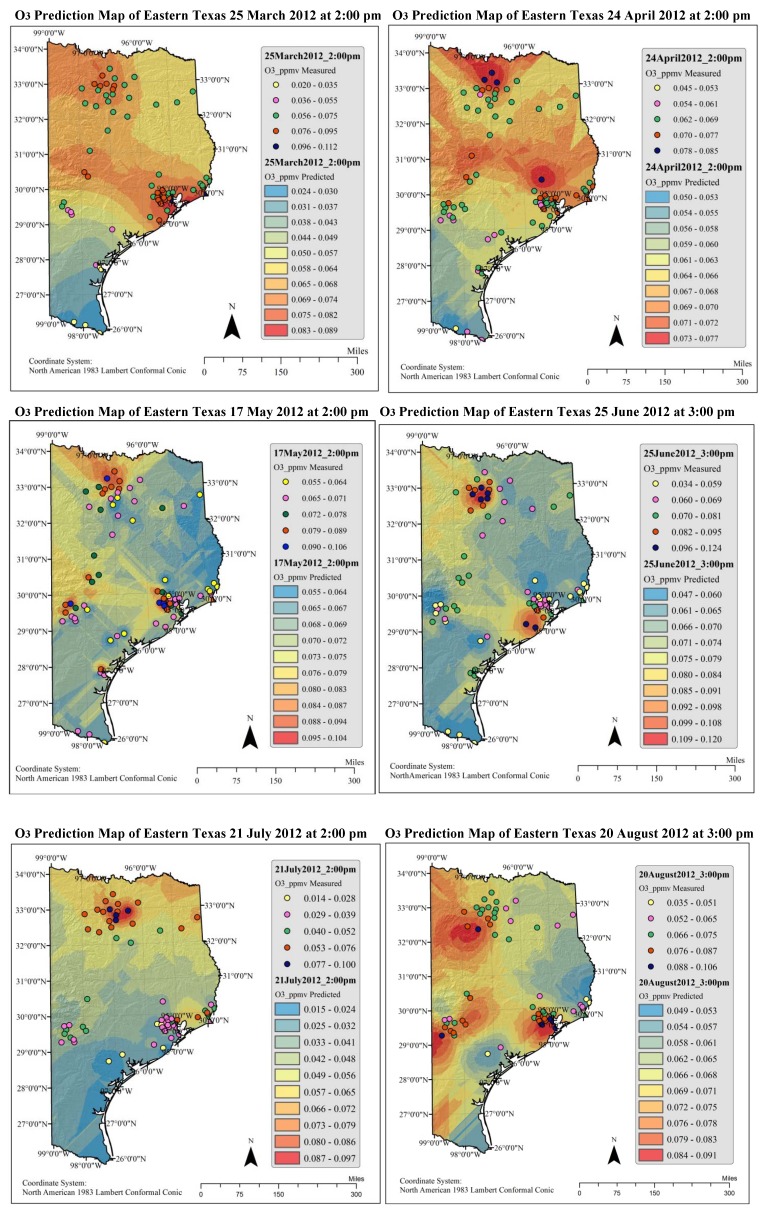
Produced pollution maps of eastern Texas for the studied hours.

Ozone pollution maps are presented in Figure 6. The maps were originally raster files that were later exported to jpeg images with a spatial resolution of 1 kilometer. To facilitate the analysis and other processing, actual results were stored in a gesostatistical (GA) layer. For the purpose of better visual understanding and producing maps of smaller cell sizes (1,000 meters spatial resolution), GA layers were later exported into raster file formats. Data presented in Figure 6 indicate that the predicted concentration of O_3_ were maximal in urban regions and also extended to suburban and rural areas. The possible reason for this observation could be attributed to more anthropogenic emissions (vehicular traffic and industrial emanations) in urban areas. This indicates that the urban population is relatively more exposed to O_3_ pollution than the rural population. The higher concentrations of O_3_ were observed during the late afternoon hours. The concentrations shown in red indicate that they are locally segregated and in all times confined to same locations. It may be evidence that the O_3_ precursors (hydrocarbons and NO_x_) could be locally generated and the higher afternoon temperatures might have caused synergism [11]. From all the examined hours, the concentrations on 25 June 2102 at 3:00 pm have elevated according to US EPA 1997 National Ambient Air Quality Standard (NAAQS), maximum hourly average concentration of 0.12 ppmv. Most of the maximum concentrations are nearer to 0.11 ppmv. 

**Figure 7:**
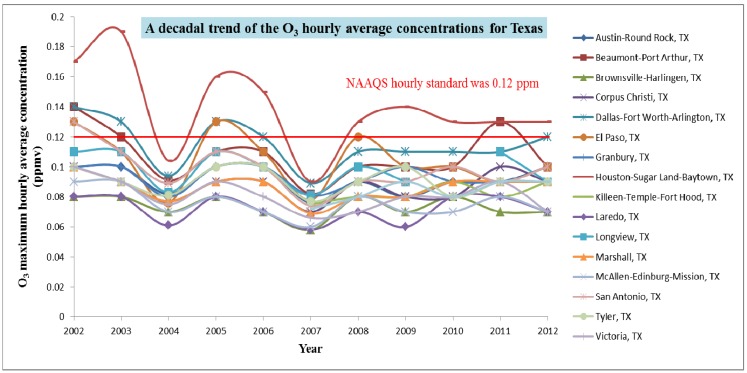
Air quality statistics- Maximum hourly average trend of Ozone in Texas for the last decade (data were obtained from US EPA [28]).

From the trend analysis in Figure 7, it is evident that the areas of Houston-Sugar Land-Baytown, Dallas-Fort Worth-Arlington, El Paso, Beaumont-Port Arthur have violated the EPA NAAQS standard. Kriging generated exposure estimates also show the same vulnerable regions (regions in west are excluded in this study).

**Figure 8:**
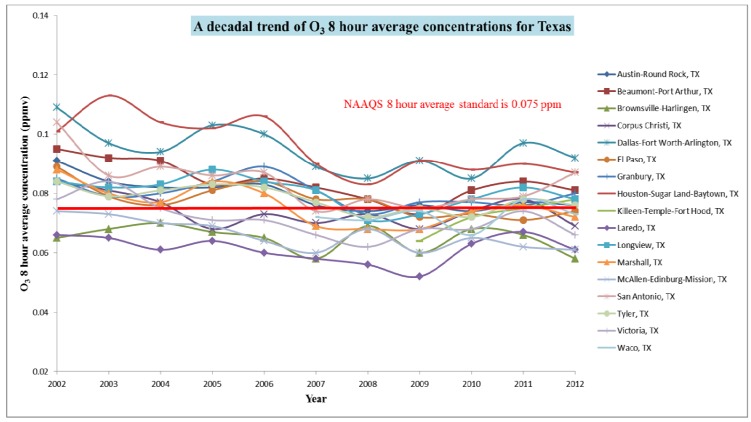
Air quality statistics- Maximum 8 hour average trend of Ozone in Texas for the last decade (data were obtained from US EPA [28]).

O_3_ falls under “definite risk” category (respiratory and cardiovascular effects) when it surpass the 0.08 ppm average for 8 hour [29]. Data shows that most of the regions (Houston-Sugar Land-Baytown, Dallas-Fort Worth-Arlington, Beaumont-Port Arthur, San Antonio, and Longview) in Texas have violated the NAAQS standard for ozone. The standard was toughened to 0.075 ppm as promulgated in 2008 [30], which pushed more regions into non-attainment status for violating the standard. It can be observed in Figure 8 that most of the cities have violated the O_3_ standard (the red line) in the first half of decade, and the trend appeared to be decreased steadily until 2009; again it started rising since then. The trend indicates that the population is frequently exposed to the harmful levels of volatile organics, NO_X_, CO, and O_3_. 

### 3.4. Cross Validation Results

Prediction error statistics presented in Table 2 indicate that three conditions discussed in cross validation methodology were met. For all the studied hours of spring and summer 2012, the mean prediction error was close to 0, the root mean-standardized-prediction error was close to 1, and the root mean square error and average standard errors were small. Although the data locations appeared to be clustered spatially in Eastern part, they generally covered most of the area (Figure 2), and prediction error statistics supports this fact (Table 2). Statistical error calculations revealed that the geographical scale and distribution of spatial data were in agreement. 

**Table 2:** Prediction error statistics for the selected hours of spring and summer 2012.

Prediction Errors	25 March 2012 at 2:00 pm	24 April 2012 at 2:00 pm	17 May 2012 at 2:00 pm	25 June 2012 at 3:00 pm	21 July 2012 at 2:00 pm	20 August 2012 at 3:00 pm
Samples	72 of 72	81 of 81	88 of 88	79 of 79	68 of 68	67 of 67
Mean	−0.0000078	0.0000022	−0.000166	−0.0000385	0.000407	0.000306
Root-Mean-Square	0.00825	0.004823	0.006229	0.00915	0.00876	0.00956
Mean Standardized	−0.00572	0.000444	−0.02046	−0.00914	0.0270	0.0207
Root-Mean-Square Standardized	0.829	0.917	0.714	1.093	1.099	1.047
Average Standard Error	0.0102	0.00527	0.00977	0.0105	0.0084	0.00927

## 4. Conclusions

Pollution mapping is a time consuming process that requires the intense efforts of scientists to think spatially, geographically, technically, and statistically to produce an accurate prediction map. To understand and solve a problem, particularly in the geo-environmental sciences, broad interdisciplinary knowledge of basic sciences, environmental science, geospatial statistical analysis, GIS, and analytical aptitude are necessary requirements. The present study was designed to predict and map ozone pollution in the Eastern region of the State of Texas, USA. Study data demonstrated that there was a spatiotemporal distribution of O_3_ with most episodes occurring during the late afternoon hours and mainly in the urban areas. The Kriging methodology appeared to be a very useful tool to successfully predict the tropospheric O_3_ pollution in our study. The technical approach of GIS and its application to monitor adverse environmental phenomena appears to be an essential decision-support tool for both environmental and human health protection. As conventional methods of data collection, analysis, and interpretation of data are time-consuming, continuous monitoring and digitally processing pollutant data in a timely manner can be more efficient. New methods of data collection using advanced instrumentation, data acquisition, assimilation, and analysis are needed to achieve this goal. In the case of O_3_ pollution, high resolution mapping is necessary to accurately generate concentrations at specific spatial intervals. A precise picture of ongoing phenomena can be obtained and understood, only when the monitoring network is optimized. Regulatory agencies like U.S. Environmental Protection Agency rely on the Kriging methodology and its prediction errors to optimize their air pollution monitoring networks [9]. Optimization not only facilitates the sample collection at reliable locations, but also reduces the sampling interval. Although it is difficult to place monitoring stations at each place of interest, one alternative is to collect field samples at non-monitored locations and integrate the ancillary data in modeling protocols. Generally, it is assumed that errors associated with predictions are low around locations close to the sampling sites, and error may increase as distance increases from sampling sites. The way GIS integrates and handles vast amounts of information in a common spatial framework enables us to use it as a powerful data management and decision-support tool. The complex relationships between air pollutants, their dispersion, spatial distribution, and population exposure can be better understood when the data are integrated in a common spatial framework that would otherwise be impossible to understand and make decisions. The EPA has promoted NAAQS standard for O_3_ in March 2008, the annual fourth highest daily maximum 8 hour concentration averaged over 3 years is set to 0.075 ppm. However, the metro cities of Houston-Sugar Land-Baytown, Dallas-Fort Worth-Arlington, Beaumont-Port Arthur, San Antonio, and Longview in Texas have severely suffered from O_3_ pollution. EPA again reconsidered the O_3_ 8 hour standard in order to protect public health and welfare, and the new final rule is set to amend the O_3_ standard to 0.070 ppm [31]. The new rule may push more regions in Texas into non-attainment status. 
